# Supplementary material for: Polyketide synthase gene clusters in the ericoid mycorrhizal fungus Oidiodendron maius: genome-wide prediction, expression profiling, and functional characterization of knockout mutants
Source: Front Plant Sci. 2026 Sep 16;17:1924430. doi: 10.3389/fpls.2026.1924430 (PMC13623903; doi:10.3389/fpls.2026.1924430)
Supplement: Supplementary file 1 [file SupplementaryFile1.zip › Supplementary Materials/Supplementary Folder 1.zip!/Oidma1_AssemblyScaffolds_Repeatmasked/knownclusterblast/cluster25/jgi.p_Oidma1_166054_mibig_hits.html]

| MIBiG Protein | Description | MIBiG Cluster | MiBiG Product | % ID | % Coverage | BLAST Score | E-value |
| --- | --- | --- | --- | --- | --- | --- | --- |
| AGO65986.1 | putative\_monooxygenase | BGC0000992 | Fusaridione\_A\_biosynthetic\_gene\_cluster | 40 | 97.7728285078 | 277 | 2.8e-74 |
| AAT65719.1 | monooxygenase | BGC0000629 | Aflatrem\_biosynthetic\_gene\_cluster | 34 | 95.7683741648 | 227 | 4.3e-59 |
| AAK11530.1 | PaxM | BGC0001082 | Paxilline\_biosynthetic\_gene\_cluster | 36 | 82.1826280624 | 224 | 2.8e-58 |
| CBX87031.1 | BcBOA8\_protein | BGC0001243\_c2 | Botryenalol\_/\_botrydial\_/\_dihydrobotrydial\_/\_botryendial\_/ | 35 | 95.991091314 | 222 | 1.1e-57 |
| BAM84049.1 | FAD-dependent\_monooxygenase | BGC0001260 | Terpendole\_E\_biosynthetic\_gene\_cluster | 33 | 92.8730512249 | 214 | 2.9e-55 |
| EAU29811.1 | predicted\_protein | BGC0001400 | Citreoviridin\_biosynthetic\_gene\_cluster | 34 | 82.85077951 | 214 | 2.9e-55 |
| ANY57883.1 | PenE | BGC0001372 | Penigequinolone\_biosynthetic\_gene\_cluster | 34 | 82.4053452116 | 207 | 3.5e-53 |
| AAW88511.1 | FAD\_dependent\_monooxygenase | BGC0001081 | Lolitrem\_biosynthetic\_gene\_cluster | 36 | 83.9643652561 | 203 | 6.7e-52 |
| EAL89233.1 | FAD-dependent\_monooxygenase\_(PaxM),\_putative | BGC0000129 | Pyripyropene\_biosynthetic\_gene\_cluster | 36 | 85.9688195991 | 201 | 1.5e-51 |
| CBF87267.1 | FAD-dependent\_monooxygenase,\_putative\_(AFU\_orthologue;\_AFUA\_8G02380) | BGC0000680\_c2 | Austinol/dehydroaustinol,\_locus\_1\_biosynthetic\_gene\_cluster | 31 | 97.995545657 | 191 | 2.6e-48 |
| CCH26290.1 | putative\_FAD-dependent\_monooxygenase | BGC0001158 | Bikaverin\_biosynthetic\_gene\_cluster | 33 | 85.5233853007 | 189 | 1e-47 |
| ACZ57545.1 | predicted\_monooxygenase | BGC0000046 | Depudecin\_biosynthetic\_gene\_cluster | 29 | 89.5322939866 | 160 | 3.8e-39 |
| AAP85357.1 | putative\_monooxygenase | BGC0000233 | Hedamycin\_biosynthetic\_gene\_cluster | 25 | 86.6369710468 | 90 | 6.3e-18 |
| AEE65479.1 | oxidoreductase | BGC0000223 | Fluostatin\_biosynthetic\_gene\_cluster | 26 | 83.5189309577 | 88 | 2.4e-17 |
| AME18017.1 | epoxidase | BGC0001378 | Tiancimycin\_biosynthetic\_gene\_cluster | 27 | 79.9554565702 | 83 | 7.7e-16 |
| AMK92570.1 | FAD-dependent\_oxidoreductase | BGC0001377 | Uncialamycin\_biosynthetic\_gene\_cluster | 26 | 76.8374164811 | 69 | 8.8e-12 |
| EWM63055.1 | monooxygenase | BGC0000679 | Diazepinomicin\_biosynthetic\_gene\_cluster | 31 | 38.7527839644 | 67 | 5.7e-11 |
| AEO12717.1 | flavin\_monooxygenase | BGC0001334 | Cladoniamide\_biosynthetic\_gene\_cluster | 25 | 97.1046770601 | 61 | 3.1e-09 |
| AKQ20698.1 | monooxygenase | BGC0001337 | Lazarimide\_biosynthetic\_gene\_cluster | 23 | 99.5545657016 | 56 | 1.3e-07 |
| AEF32093.1 | monooxygenase | BGC0001333 | BE-54017\_biosynthetic\_gene\_cluster | 25 | 77.9510022272 | 55 | 2.2e-07 |
